# Editorial: Editor’s Pick 2021: Highlights in Cell Death and Survival

**DOI:** 10.3389/fcell.2022.887688

**Published:** 2022-05-09

**Authors:** You-Wen He, Craig M. Walsh

**Affiliations:** ^1^ Department of Immunology, Duke University School of Medicine, Durham, NC, United States; ^2^ Department of Molecular Biology and Biochemistry, Sue and Bill Gross Stem Cell Research Center, University of California, Irvine, Irvine, CA, United States

**Keywords:** cell death, autophagy, survival, T lymphocyte, antitumor immunity, immunotherapy

The Cell Death and Survival section of the Frontiers in Cell and Developmental Biology was launched in year 2013 to serve as an open-access venue for outstanding research and commentary in the field. This section has witnessed a dramatic increase in manuscript submission and publication of high-quality original research manuscripts and reviews addressing important Research Topic in cell death and survival in the last year. The published papers in our section cover a broad swath of topics in the area cell death and survival, from fundamental molecular and cellular biology to clinical application of cell death and survival principles. To give the broad readership of this journal an overview of the activities in the section, a Research Topic called Editor’s Pick for section editors to highlight significant contributions to the field provides a brief introduction to some of the cutting edge research and influential reviews in our section. Due to a limit on the number of papers allowed for Editor’s pick, many high-quality research manuscripts and insightful reviews published in our section deserve as much attention. We highlight the following publications to showcase the breadth and depth of these scholarly contributions to our section. Moreover, we thank all contributors to our section and know that the growth in Frontiers in Cell and Developmental Biology has been driven by these important contributions.

The function of the Bcl-2 family member Bok (Bcl-2 related ovarian killer) remains mysterious. Although Bok was initially classified as a pro-apoptotic protein due to its sequence homology to Bax and Bak, accumulating evidence suggests that Bok is involved in many other cellular functions (Naim and Kaufmann). Szczesniak et al. used TurboID-mediated proximity labeling to probe the Bok interactome. They reported that Bok interacts with, and is proximal to proteins involved in various cellular functions such as mitochondrial fission, endoplasmic reticulum-plasma membrane junctions, and surprisingly, Mcl-1. The physical and functional interactions between Bok and Mcl-1 were further verified. Importantly, these authors showed that Bok interactome is distinct from those of Mcl-1 and Bak. This report has provided important functional insights into Bok in apoptotic and non-apoptotic intracellular processes and will instigate additional research into this important area.

The serine/threonine kinase 35 (STK35) was known to be upregulated in colorectal cancer (CRC) but its function in tumorigenesis was unknown (Capra et al., 2006). Yang et al. reported that STK35 expression is negatively associated with CRC patient survival. In a series of gain- and loss-of-function experiments, the investigators demonstrated that STK35 inhibits tumor cell apoptosis and increases glycolysis by activating the AKT pathway. Importantly, STK35 overexpression renders CRC tumors resistant to 5-FU chemotherapy. This study suggests that STK35 may be used as a prognostic biomarker and therapeutic target for CRC diagnosis and treatment.

Immune checkpoint inhibitors such as anti-PD-1 and PD-L1 antibodies have revolutionized cancer therapy by overcoming immune exhaustion (Lei et al.). Zhang et al. investigated the expression and prognostic value of a newly identified immune checkpoint receptor Human endogenous retrovirus-H Long terminal repeat-Associating protein 2 (HHLA2) in 22 types of solid tumors. HHLA2 was reported to be expressed in all 22 tumors at the mRNA level and 12 types of these tumors at the protein level. Furthermore, HHLA2 was found to have the highest transcript levels in kidney clear cell carcinoma (KIRC) and was positively correlated with better survival and high CD8 expression in this group of patients. This study reveals a potential immunotherapeutic target for KIRC treatment.

Cellular senescence, a stable exit from the cell cycle in response to different stresses and cellular replication, is tightly linked to physiological and pathological processes such as aging and cancer (Herranz and Gil, 2018). Premature senescence may contribute to early thymus involution and immune dysregulation in Down Syndrome patients (Marcovecchio et al., 2021). Yu et al. found that transient ectopic expression of a repressive epigenetic modulator, DNA methyltransferase 3-like (DNMT3L), delays the premature senescence progression in mouse embryonic fibroblasts. The authors further investigated molecular mechanisms underlying DNMT3L-mediated chromatin surveillance through epigenetic regulation. This study is significant as a new epigenetic reinforcement strategy may be used for overcoming premature senescence in a variety of diseases.

Hypoxic ischemic encephalopathy (HIE) causes severe disability and death in ∼400,000 newborns worldwide each year (Victor et al., 2021). Xiong et al. studied the molecular network regulating neuronal cell death after HIE and identified a novel long non-coding RNA (lncRNA) Vi4 (TCONS00044054) as a key regulator of neuron survival and apoptosis after HIE. Furthermore, the investigators demonstrated that a regulatory network consisting of Vi4, miR-185-5p, and the target gene Igfbp3 plays an important role in neuronal death and survival after HIE, highlighting potential strategies for therapeutic intervention of HIE.

Bioactive compounds including baicalein, baicalin, wogonin and wognoside from the roots of the medicinal plant *Scutellaria baicalensis* have potent antitumor activities (Banik et al., 2022). Huang et al. studied the antitumor activities of baicalein and baicalin in melanoma cells. These authors showed that baicalein and baicalin inhibits proliferation and induces apoptosis and senescence in melanoma cells. Mechanistically, baicalein and baicalin were found to inhibit tumor cell glucose uptake and metabolism by downregulating the mTOR/HIF-1α signaling pathway. This study suggests a potential for baicalein and baicalin as novel chemotherapy drugs.

Human aldo-keto reductase 1B10 (AKR1B10) may play roles in gastrointestinal (GI) tract function and be involved in GI cancers and inflammatory bowel diseases (Endo et al., 2021). Wang et al. characterized mice lacking AKR1B8, the mouse ortholog of human AKR1B10. The investigators found that the integrity of the intestinal epithelial barrier in AKR1B8 deficient mice was severely disrupted. Furthermore, innate and adaptive immune cell populations in the colon of AKR1B8 deficient mice were dramatically altered in composition and function. This study suggests that AKR1B8 is vital to the maintenance of intestinal epithelial barrier and normal immunity within the colon.

Autophagy plays an essential in regulating T lymphocyte survival and homeostasis (McLeod et al., 2012). Xia et al. investigated the T cell compartment in Beclin 1/Atg6 conditional knockout mice and found a diminished naïve T cell population, increased effector T cells and MDSCs, and severe colitis in aged mice. Interestingly, the reduced population of naïve T cells was rescued by crossing the conditional Beclin 1/Atg6 KO mice onto a TCR transgenic background, which also led to a normalized population of effector T cells. These data provide support for context-dependent roles of Beclin1/Atg6 in T lymphocyte survival and differentiation.

Intratumoral administration of immunotherapies has the potential to maximize immune activating capability and minimize systemic toxicities of immunotherapeutic agents (Melero et al., 2021). Hu et al. tested the antitumor activities of intratumorally injected IL-27 using recombinant adeno-associated virus (rAAV)-based delivery. The authors demonstrated that intratumoral injection of rAAV-IL-27 led to strong antitumor activities in several mouse tumor models. The investigators further showed that IL-27 induces infiltration of CD8^+^ T cells and CXCR3 expression and imparts synergistic antitumor activities with anti-PD-1 administration or adoptively transferred T cells. These interesting preclinical data suggest that intratumoral delivery of IL-27 may be useful alone or in combination with other therapies for cancer treatment.

In addition to the above nine original research papers, we would like to highlight several insightful reviews published in our section. One example focused on ionizing radiation as a major cancer treatment modality, highlighting its ability to induce ferroptosis and alter fatty acid metabolism in tumor cells. Yuan et al. reviewed the pathways between ionizing radiation and ferroptosis and the critical roles of fatty acid metabolism in radiation-induced ferroptosis in tumor cells. Importantly, the authors provided thoughtful perspectives on the implications of the interplay between fatty acid metabolism, ferroptosis and ionizing radiation for future clinical development of novel cancer treatment modalities. Many clinical drugs have known cardiotoxicities due to their ability to induce various forms of cell death in cardiomyocytes. Ma et al. first reviewed the different forms of cell death including apoptosis, autophagy, necrosis, necroptosis, pryoptosis and ferroptosis in cardiomyocytes. These authors further analyzed the underlying mechanisms of cardiomyocyte death induced by three major classes of clinical therapeutics: anti-tumor, anti-diabetic, and anti-viral drugs. This review has offered both an excellent theoretical overview and practical guidance for physicians in therapeutic application of these clinical drugs. In a more targeted review by Zhai et al., the roles of one specific form of cell death, ferroptosis, in cardiomyopathy are discussed in depth. The potential for utilizing the ferroptosis pathway as a diagnostic and therapeutic target for patients suffering from cardiomyopathy was carefully analyzed. T lymphocytes are essential for the antitumor efficacies of immunotherapies. However, primary and acquired resistance to immunotherapies are common and may be at least partially caused by T cell dysfunction in the tumor microenvironment of cancer patients. Zhang et al. provided a comprehensive update on the molecular mechanisms of T cell dysfunction and exhaustion in the tumor microenvironment and potential strategies to overcome these defects. In summary, the above reviews have provided timely information and thoughtful insights in a wide array of topics with high scientific and clinical significance.

## References

[B1] Banik K. Khatoon E. Harsha C. Rana V. Parama D. Thakur K. K. (2022). Wogonin and its Analogs for the Prevention and Treatment of Cancer: A Systematic Review. Phytother. Res. 31, 1–27. Epub 2022/02/02. 10.1002/ptr.7386 35102626

[B2] Capra M. Nuciforo P. G. Confalonieri S. Quarto M. Bianchi M. Nebuloni M. (2006). Frequent Alterations in the Expression of Serine/Threonine Kinases in Human Cancers. Cancer Res. 66 (66), 8147–8154. Epub 2006/08/17. 10.1158/0008-5472.CAN-05-3489 16912193

[B3] Endo S. Matsunaga T. Nishinaka T. (2021). The Role of AKR1B10 in Physiology and Pathophysiology. Metabolites 11, 11. Epub 2021/06/03. 10.3390/metabo11060332 PMC8224097 34063865

[B4] Herranz N. Gil J. (2018). Mechanisms and Functions of Cellular Senescence. J. Clin. Invest 128 (128), 1238–1246. Epub 2018/04/03. 10.1172/JCI95148 29608137 PMC5873888

[B5] Marcovecchio G. E. Ferrua F. Fontana E. Beretta S. Genua M. Bortolomai I. (2021). Premature Senescence and Increased Oxidative Stress in the Thymus of Down Syndrome Patients. Front. Immunol. 12, 669893. Epub 2021/06/19. 10.3389/fimmu.2021.669893 34140950 PMC8204718

[B6] McLeod I. X. Jia W. He Y.-W. (2012). The Contribution of Autophagy to Lymphocyte Survival and Homeostasis. Immunol. Rev. 249, 195–204. Epub 2012/08/15. 10.1111/j.1600-065x.2012.01143.x 22889223 PMC3602971

[B7] Melero I. Castanon E. Alvarez M. Champiat S. Marabelle A. (2021). Intratumoural Administration and Tumour Tissue Targeting of Cancer Immunotherapies. Nat. Rev. Clin. Oncol. 18, 558–576. Epub 2021/05/20. 10.1038/s41571-021-00507-y 34006998 PMC8130796

[B8] Victor S. Rocha-Ferreira E. Rahim A. Hagberg H. Edwards D. (2021). New Possibilities for Neuroprotection in Neonatal Hypoxic-Ischemic Encephalopathy. Eur. J. Pediatr. Nov. 24. Epub 2021/11/26. 10.1007/s00431-021-04320-8 PMC8897336 34820702

